# Dignity in relationships and existence in nursing homes’ cultures

**DOI:** 10.1177/09697330211041739

**Published:** 2022-07-08

**Authors:** Arne Rehnsfeldt, Åshild Slettebø, Vibeke Lohne, Berit Sæteren, Lillemor Lindwall, Anne Kari Tolo Heggestad, Maj-Britt Råholm, Bente Høy, Synnøve Caspari, Dagfinn Nåden

**Affiliations:** Western Norway University of Applied Sciences, Faculty of Health and Social Sciences, Department of Health and Caring sciences; University of Agder, Faculty of Health and Sport Sciences, Department of Health and Nursing Sciences, Norway; Oslo Metropolitan University, Faculty of Health Sciences, Department of Nursing and Health Promotion, Norway; Karlstad Univeristy, Faculty of Health, Science and Technology, Department of Health Sciences; VID Specialized University, Faculty of Health Studies, Department of Nursing, Norway; Western Norway University of Applied Sciences, Faculty of Health and Social Sciences, Department of Health and Caring sciences; Randers Regional Hospital, Denmark; Oslo Metropolitan University, Faculty of Health Sciences, Department of Nursing and Health Promotion, Norway

**Keywords:** Caring culture, dignity, dignity in relationships, ethics, existence, existential, hermeneutics

## Abstract

**Introduction:**

Expressions of dignity as a clinical phenomenon in nursing homes as expressed by caregivers were investigated. A coherence could be detected between the concepts and phenomena of existence and dignity in relationships and caring culture as a context. A caring culture is interpreted by caregivers as the meaning-making of what is accepted or not in the ward culture.

**Background:**

The rationale for the connection between existence and dignity in relationships and caring culture is that suffering is a part of existence, as well as compassion in relieving suffering, and ontological interdependency.

**Aim:**

To describe different expressions of dignity in relationships and existence in context of caring cultures from the perspective of the caregivers.

**Research design:**

The methodology and method are hermeneutic. The method used was to merge the theoretical preunderstanding as one horizon of understanding with empirical data.

**Participants and research context:**

Focus group interviews with caregivers in nursing homes.

**Ethical considerations:**

The principles of the Helsinki Declaration have been followed to, for example, preserve self-determination, integrity, dignity, confidentiality and privacy of the research persons.

**Findings:**

Data interpretation resulted in four themes: Encountering existential needs that promote dignity in a caring culture; To amplify dignity in relationships by the creative art of caring in a caring culture; Violation of dignity by ignorance or neglect in a non-caring culture and The ethic of words and appropriated ground values in a caring culture.

**Discussion:**

Dignity-promoting acts of caring, or dignity-depriving acts of non-caring are adequate to see from the perspective of dignity in relationships and existence and the caring culture.

**Conclusions:**

Dignity in relationships seems to touch the innermost existential life, as the existential life is dependent on confirmation from others.

## Introduction

Expressions of dignity as a clinical phenomenon in nursing homes as expressed by caregivers were investigated. In the ‘naïve reading’ of the interview data, a coherence could be detected between the concepts and phenomena of existence, dignity in relationships and caring culture as a contextual factor with ethos as its ontological roots.

Dignity guarantees have been implemented in the Scandinavian countries. Still, the Danish minister for elder care says that she will restore dignity in the care of the older people.^1^ Not least, the COVID-19 pandemic has shown that restoring dignity is an urgent matter where older people have been unduly affected by severe disease or death for various reasons. In a Scandinavian project, residents, relatives and caregivers were interviewed to understand dignity as a clinical phenomenon. The interviews with the caregivers in this project were performed as recurrent focus group interviews at five nursing homes in Scandinavia, with at least three interviews with each focus group in Norway, Denmark and Sweden.

## Background

In former results of this research project, the connection between relationships, dignity, existence and caring culture can be seen in different forms, for example, the relationship between vulnerability, existence and unfavourable care contexts.^2^ Another aspect is that paternalism reduced the sense of freedom of the residents.^3^ Yet another perspective that connects existence to dignity in relationships and caring culture is, in congruence with (Sæteren et al.), to promote personal life space ontologically, relationally and physically to stimulate health resources^4^ and give meaning to the world.^2^ If the caregivers are committed to the residents this may happen, and also that caring cultures can grow, characterised by at-homeness, where each resident is seen as a unique person.^5^ Expressions of dignity, especially as an ontological concept related to praxis concepts,^6^ is exemplified below, where we can see that the authors relate dignity as an ontological concept to different forms of relationships in praxis.

### Dignity in relationships

Dignity is upheld in social relationships.^7^ In line with this, Eriksson proposes that a caring communion honours the fundamental value of dignity.^8^ A caring communion in a caring culture has an atmosphere of invitation and charity, the goal of which is to preserve the dignity of the patients by allowing them to rest in their present existential situation.^9,10^

Dignity is also an aspect of humaneness as respect for others is something that is learnt.^11^

Different caring acts including, for example, individualised care, showing respect, advocacy and listening sensitively^12^ can uphold dignity.

Relative dignity is constituted in social and cultural experiences.^13^

### Existence from a caring science perspective

Closely related to ‘dignity in relationships’ is the description of existence in caring science. Existence, or the meaning of being human, is a core concept of the three Scandinavian theorists and scholars Eriksson, Martinsen and Dahlberg.^14^

Eriksson finds that suffering is central to human existence, where people experience being authentic when they open to suffering. Also, not to be confirmed in one’s humaneness by others is an utterance of ‘dying’ by suffering. Another form of confirmation is to be confirmed in one’s dignity by others, as a way towards finding meaning in suffering.^10^

Martinsen sees vulnerability and interdependence as natural or ontological human characteristics that are connected to each other by, for example, compassion.^15^ The lived experience of being vulnerable is an existential experience.

Existential life alludes to priorities and values of life, relationships with others, but also love and death. When these existential matters are in the foreground of life experiences, they are a basis for forming a new understanding of life. A prerequisite for existential care related to the existential life of the patient^16^ is, according to Lindström et al., that a caring communion takes place.^9^

It is important to focus on the inner world of the older persons as an existential aspect as Hall and Høy have shown, in that clinical nurses caring for older persons perceive caring as mainly related to upholding dignity by seeing the uniqueness of the other.^17^ The concept ‘to be seen upon’ is also relatable to both ‘acquaint for the inner world’ and the ‘uniqueness’ of the other as Harstade et al.^18^ state.

### Caring culture

Seen from the perspective of relatives, a caring culture emerges when the caring culture mirrors the ethos or ontology of human dignity. Also, ethical acts reflect the meaning-making of the caring culture by individual caregivers.^19^ In accordance with Rytterstöm et al., a caring culture may be hidden in the ward, but is still interpreted by individual caregivers as the meaning-making of what, for example, is accepted or not in ward culture.^20^

Slow caring can be a part of the caring culture. Lohne et al. show that slowing down as a mode of existing in caring allows the quality of proper space and time to be at hand in caring for a fellow human being. Quality in caring is pronounced before quantity in this caring attitude.^21^ The analogue concept ‘slow nursing’ honours the idea that caring is the essence of nursing. Slow nursing can contribute to valuing persons with dementia in their personhood by the caring relationship.^22^

Relating to the idea that the basic values of caring reveal themselves in a caring culture, Frilund, Eriksson and Fagerström plead that without a connection between possibilities of providing dignified care in a caring communion based on ethical values and an ethical discussion, adequate care of older persons may not be guaranteed.^23^ This shows that ethical discussions provide a possibility to appropriate the social aspect of dignity. Also, according to Dauwerse, van der Dam and Abma, there is a need for support in ethical matters of an everyday nature in a climate of dialogue.^24^ On the opposite end, caregivers working in a nursing home often meet and must handle difficult ethical situations where the culture may not support the ideals of dignity and autonomy.^7^

In a caring culture where caregivers meet persons with newly diagnosed dementia as equal human beings with an attitude of understanding and warmth, it is existentially crucial for them to experience dignity.^25^

In summary, as the literature review has shown the rationale for the connection between existence and dignity in relationships and caring culture is that suffering is a part of existence, as well as compassion in relieving suffering, and ontological interdependency. A caring communion is characterised by charity and invitation to a caring culture with a living ethos.

## Aim

The aim is to describe different expressions of dignity in relationships and existence in context of caring cultures from the perspective of the caregivers.

## Hermeneutics as methodology

The philosophical ground or methodology of the hermeneutic method is Gadamer’s philosophical reasoning on hermeneutics.^26^

## Method

The hermeneutic method used was to merge the theoretical preunderstanding as described above as one horizon of understanding with empirical data from focus group interviews with caregivers in nursing homes to reach understanding by a fusion of horizons.^26^

### Setting and participants

The data originate from five nursing homes in Scandinavia: two in Norway, two in Sweden and one in Denmark. On average, about eight participants were present at each occasion. An interview guide was used with themes for three gatherings with each focus group. At one of the nursing homes, four gatherings were held where data from the former meetings were summarised and discussed at meeting number 4.

Participants in the focus groups were caregivers with different professions except medical doctors. The duration for each session was about 1 hour and it was tape-recorded.

### Interpretation of data

Interpretation of the data was inspired by the concepts aim, seeking and closing. The aim is decided by a caring science theoretical position. The seeking is to search for clinical expressions of the theoretical base. In the closing phase, the ontological, ethical and clinical understandings are interweaved into a new comprehensive understanding.^27^ To achieve this, a table with four columns was used. Examples of data interpretation are presented in Appendix 1.

Each focus group interview was analysed by the whole research team in a reflexive manner.

### Ethical considerations

As a part of a larger Scandinavian project, this subproject was approved by ethical committees in the respective participating country and the Norwegian Social Science Data Services. Participation in this study was voluntary. The participants were informed orally about the project, voluntariness and the option to withdraw, and gave written consent by signing forms.

The principles of the Helsinki Declaration have been followed, for example, to preserve self-determination, integrity, dignity, confidentiality and privacy of the research persons.^28^

## Findings

### Encountering existential needs that promote dignity in a caring culture

Existential needs can be seen in terms of a life that is worth living and fun by doing cultural activities. A culture that allows for this can enhance worthwhile living. Another expression of how to meet the existential needs is to be seen in one’s life history where dignity is embedded and cared for when seen.

Caregivers talked about a system of primary nurses for each resident to learn more about the resident’s life. In addition, to be able to show love to meet the resident with closeness and caring so that the resident can live a life with full respect, love and care requires a mood of meeting existential needs. Another form of existence is to have their spiritual needs satisfied and being able to practice their religion.

An existential need is to feel of age, that is, to be cared for as an adult, despite one’s physical condition. ‘I have been disempowered, no one listens to me’, said a woman with dementia according to a caregiver.

An expression of safety as an existential need is when a nurse saw that a resident felt much safer with her, so the caregiver decided to visit the resident every day at work. This also made the resident’s everyday life easier.

### To amplify dignity in relationships by the creative art of caring in a caring culture

This theme shows how creativity mirrors the art of caring and how artistic caring can create results through being aware of existential needs and enhancing dignity in caring for the residents.

A former professional caregiver would not leave the chair she was sitting in and would not go to the toilet. When the caregivers appealed to her professional knowledge, she stood up and walked to the toilet. Another expression of the art of caring is to read the eyes of a resident while, for example, giving tactile massage, to see if he or she likes it or not.

Preserving dignity can be to find solutions on things and become a ‘master of solutions’, for example, to bring fruit into the shower because the resident liked fruit. Another example was to bring a resident to a football match with the team from his home district, although the caregiver ‘hated’ that specific team. The art of caring can also take place when noticing ‘small things’ that often take place in everyday situations.

### Violation of dignity by ignorance or neglect in a non-caring culture

This theme is related to suffering induced by care^10^ and non-ethical performance individually and in a culture, by the way dignity is violated by ignorance and neglect. This could be related to power relationships between staff and residents. For example, when the dignity of residents is violated, they become silent.

Not criticising behaviours of ignorance by colleagues when, for example, they do not answer calls from residents is a way to contribute to ignorance by not taking responsibility. Another example of this ignorance is when a person wanted a special breakfast and her contact person of the staff fought for it, but the rest of the staff resisted the idea because it was not a rational way to work.

Dignity is at risk of being violated when new knowledge is not properly used as the examples below show. When caregivers have attended in-service training, nothing happens to implement this in the caring situation because everyone waits for someone else to do it. When we have ‘development days’ on the ward, we say that we will do such and such, but we ‘should need to have that discussion once a week to get the thoughts into our heads, otherwise you let it go’.

Undignified care can also appear due to attitudes towards old persons. When, for example, someone feels bad, the staff backs off and runs away as far as possible, and says ‘Oh God, what is he up to’? ‘We focus a lot on external things and are wary of the inner’.

In addition, expectations in the culture of the ward can enhance not taking proper responsibility for dignified care. For example, when sitting bedside by a resident you get a bad conscious for not participating in the practical work. To overcome this bad conscious, you need to be confirmed by someone else who says, ‘you are not lazy’ but do just as much as me.

Focusing on outer aspects of care can violate inner dignity. An example is the report situation in the afternoon, where there is much discussion of whether the residents had showered or if their stomach is in order, but not about how they are feeling. ‘But at the same time, I think both sides are necessary. You cannot walk around all day with people who are philosophical’.

Other examples of neglect are not to dress the residents properly for meals. Neglect of the residents can also take place in relation to their relatives, and one runs over to the residents to satisfy the relatives. We sometimes talk about ‘relative-infusion’ and ‘relative penicillin’ not because the residents need this, but because of the relative’s wish. Relatives can question the care of their older relatives, and even examine them themselves (e.g. for obstipation). Another example of neglect is to seem as busy as possible as a caregiver, as this is a modern trend.

### The ethic of words and appropriated ground values in a caring culture

The ethic of words^29^ relates both to dignity in relationships and existence. Badly chosen words can cause suffering, humiliation and loss of dignity. Appropriation of ground values means to make these values one’s own as an inner appropriation and to apply them in caring.^6^ The ethic of words means to choose words carefully when relating to another person, where words can even bring relief from suffering. The data show this in different ways, for example, when the tone of language between caregivers themselves and towards residents is contagious in the working environment. When you choose collective words like ‘the demented people’, their dignity is put at risk. Also, words should be avoided that may diminish the residents by, for example, putting them in a child’s position and using a word like bib instead of a more dignifying word like apron. Another example is when a person with cancer is met by a young caregiver with the words ‘Oh dear are you still alive’.

Ground values are seen here as the ontological value base or ethos for caring, and appropriation means that the caregivers actively use these ground values when caring for their residents. In the words of one caregiver, ‘you must live your attitudes’.

When a caregiver says that you cannot see the residents as a group, as each resident is unique, an appropriated worldview reveals itself.

To see the resources of each resident and to pronounce the importance of having background knowledge of the residents are other examples of appropriated ground values.

Other utterances of appropriated ground values are caring with the heart, which gives ‘meaning to why I am here’, as well as to recognise the human value of the fellow human being.

## Discussion

To see all the themes in the context of caring cultures is due to the preunderstanding that caring cultures always exist, either overtly or covertly, and that the culture is meaning-making to the caregivers when they interpret what is accepted or not in the institution.^20^

### Existence and existential needs related to dignity in relationships in a caring culture

To be seen in one’s life history can be related to existential concepts like being allowed a life space, to be confirmed in humanness and be valued, to be in communion^10^ and interdependence^15,30^ with committed caregivers.^31^ When primary nurses for each resident are seen as important, it can open up for expressing an ethical demand^30^ by being allowed to express one’s existential needs and suffering in a caring communion.^10^ Also, caregivers have an existential need to be allowed to show love when they have seen the ethical demand from the resident. This can be seen as an existential response to the ethical demand or, according to Løgstrup, a sign of life utterances based on ontological interdependence.^30^

When residents are disempowered by caregivers, it is a clear sign of a non-caring culture without ethical discussions^23^ and an attitude of paternalism,^3^ and an atmosphere of coldness opposite of warmth.^25^

An existential need is to feel safe in a caring communion,^10^ and this can be illustrated by the nurse who visited a resident every day because she saw that the resident felt much safer with her. This made the resident’s daily life much easier.

Another insight in utterances in the interview data was to confirm residents’ existential needs to exercise a spiritual life. This is because, in accordance with Jacelon et al., residents are seen as a unit.^11^ It can also be interpreted in terms of that the older person’s personal life space is promoted in the nursing home.

What is said above is fully in line with what Jacobsen and Sørlie say about dignity being created in relationships, and the necessity to reflect this insight back by language and caring acts towards the person cared for.^7^ For many old persons, ageing may be an ontological experience and an existentially vulnerable period of life or even a boundary situation causing suffering.^16^ This is due to losing one’s earlier existential life. Also, contextual changes like moving to a nursing home and losing one’s home is an existential transition. It could be a period where human interdependence as an ontological prerequisite of life has to be tried out, and according to Løgstrup, a person’s entire life may be in the hands of a fellow human being.^30^ It is a period of life where the concepts of caring communion and slow caring may relate to each other, as slow caring is a prerequisite for caring communion. In the caring communion, the caregiver directs no demands towards the person cared for, but only gives them an invitation to rest with the fullest possible dignity and be honoured in their present existential situation. In accordance with Eriksson, the birth of dignity can be seen in a caring communion.^8^ A caring communion accordingly stands up as an expression of interdependence where the other person can rest with full dignity, without demands and outer pressures in communion with others. In accordance with slow caring,^21^ which is a necessity if a caring communion is to take place,^8^ personhood is valued^22^ by the time and space allowed for by slow caring.^21^ In order for this to happen, the ward culture cannot ignore dignity but must honour it.

### The art of caring in a caring culture

We have seen in the data characteristics of a caring culture where the art of caring is at hand when a caring culture is characterised by an underlying ethos. The characteristics of the art of caring are, as seen in the data, creativity, being sensitive to the other person by, for example, eye contact during a caring moment, using the skills of the residents to do the ‘little extras’ and paying attention to^7^ small things that appear in everyday situations.^32^ This provides clinical and ontological evidence to Nåden’s theoretical model of nursing as an art. He says that the art of caring gives the utmost quality to the care given and always promotes growth.^33^ One example of this is to appeal to the former physiotherapist’s professional skills. One of the bearing categories in Nåden’s theoretical model is invitation and confirmation. It means to validate the fellow human. Not to invite and confirm means to not see and take the other seriously. This may lead to suffering and violation of dignity. A prerequisite for invitation and confirmation is that the caregiver shows an attitude of calmness as a sign of inner security.^33^ This calmness could be compared to the concepts ‘slow caring’^21^ and ‘slow nursing’^22^ discussed above.

### Violation of dignity in a non-caring culture

As said above, a caring culture reveals an ethos as a basis for caring communions to take place, and ethical acts reflect the meaning-making of the caring culture. Rytterström, Arman and Unosson also relate abuse to care cultures when they say that abuse of the older persons can take place if the inner world of the other is not accounted for in the caring culture.^34^ A non-caring culture is characterised here by caregivers giving meaning to the non-existent caring culture by acting on an individual basis, and not on a common ethos. Also, not to notice the inner (existential) world of the fellow human being could be an act of dignity violation.

Examples of non-caring behaviour in the results are to exercise power, neglect and ignorance towards the residents, not answering calls, running away from a disturbing resident, not dressing persons properly for meals or ignoring residents to satisfy relatives. These are examples of both concrete and existential abandonment. Concrete examples are when caregivers leave the residents on their own. Existential abandonment appears when, for example, the caregivers do not confirm the old persons, or when caregivers are not engaged in the residents and therefore are not inviting them. This could lead to suffering or even suffering of care when acts of non-care are the direct cause of suffering.^10^ Existential abandonment seems to connect to the culture of the nursing homes when, for example, practical work is more valued than sitting ‘by the bedside’ of the residents. This bedside ‘existential work’ is when there is a chance to invite a dialogue on important issues in life and thereby confirming that the caregiver sees the old person as a human being.

It seems important to accommodate to the ward culture as the findings have shown. A study by Feltrin, Newton and Willets shows that one way to fit in is to try to understand those who do not fit in and avoid maladaptive behaviours.^35^ Of course, adaptation to the ward culture could also be a positive thing that promotes dignity in the care provided to the care recipients. However, a care culture can also be oppressive, as shown in the data, where unspoken rules steer the culture. These rules are, for example, that practical work is more important than ethical work meeting existential needs or not to dare to be critical towards colleagues and thereby not take own responsibility for the care given. It becomes a form of collective irresponsibility where neglect easily may appear. On the other hand, a ward culture can be truly caring in itself as the study by Arman et al.^36^ has revealed. Roets, Poggenpoel and Myburgh have shown that aggression among staff can be both verbal and non-verbal. Non-verbal aggression is for example, to be judged by colleagues.^37^ Examples of this judging in our data are to experience resistance from colleagues when proposing dignifying acts of caring, or to have a bad conscious when sitting with a resident and not participating in ‘practical work’.

### Dignity in relationships expressed in the ethics of words and appropriated ground values in a caring culture

Eriksson describes *the ethics of words* when she says that words mirror one’s values and the tradition where one belongs. In the tradition of caring science, the words serve the essence of caring, that is, to ease suffering. The words bear witness of the ethos of caring when the caregiver dedicates the ethical words to the fellow human being in a dialogue.^29^ As seen in the findings, words have the potential to cause ‘suffering of care’^10^ and violation of dignity, and probably this is due to that the caregivers have not appropriated the ethos of caring, and that there may be a lack of ethos in the (caring) culture. To be aware of the words of caring is also a clear example of dignity in relationships, based on ontological interdependency where people form their lives together.

Eriksson says that ethos leads the words from the heart via thoughts to the hand (c.f. the head-heart-hand model for caring),^29^ and this seems evident if you look at the content in *appropriation of ground values*. Examples in the data are to see everyone as unique with specific resources, and to allow oneself to show love towards another person. Yet another mode of thinking on appropriation is when Eriksson says that any form of appropriation contains understanding, exposition and application. The exposition reflects one’s ethos, and the words chosen convey an ethical loading. Application instantly follows understanding.^38^

### Methodological considerations

Finally, to reflect a bit on methodology and the methods used, it is important to be clear on one’s disciplinary belonging to interpret and understand data from a hermeneutic point of view. This is due especially if you look at the latent content of data, that is, the meaning of data. This needs to be interpreted as it is not always overt but covert. A fusion of horizons has been achieved, not least due to common and repeated interpretation efforts that have been done in the whole research group, by discussing the interpretation several times in a reflexive manner to reach a unanimous understanding of the data.

Different cultures in the Nordic countries might have affected the results.

## Conclusion

The result shows that dignity-promoting acts of caring, or dignity-depriving acts of non-caring, are adequate to see from the perspective of dignity in relationships and existence and caring culture. The culture sets the limit and allowance for dignity-based caring and is something that is always interpreted by the caregivers in every caring act. Dignity in relationships stands out not only in the close caring relationship but also in the relationship between co-workers in a caring culture. In fact, expressions of dignity in relationships often seem to melt together or be interchangeable with utterances of existence. So, dignity in relations seems to touch the innermost sense of the existential life, as the existential life is dependent on confirmation from others in a relationship and caring communion characterised by dignity.

What is described above can be seen as an ontological caring ethics that describes the ‘what’ of ethics, and how this can be appropriated by caregivers as their ethical attitude to be applied in ethical acts of caring. In accordance with this, Eriksson says that the ultimate ethical goal is to guarantee the dignity of fellow human beings. Eriksson defines ontological ethics to search for the true essence of ethics, and not to stay in ‘technological’ rules for conducting ethics.^39^

## Appendix

**Appendix 1.** Examples of data interpretation.

What is said (aim)	What is spoken about (seeking)	Interpretation (seeking)	Theme (closing)
What caregivers often talked about is a system of primary nurses for single residents, as a way to learn more about the resident’s life.	A primary nurse whose task is to learn more about the other’s life	Can be connected to existential needs of being confirmed in one’s life, compassionate commitment by interdependence, caring communion, ethical demand to express one’s existential needs and suffering.	Encountering existential needs that promotes dignity in a caring culture
The tone of language between caregivers and residents is contagious in the working environment.	The tone of language between staff members is contagious in the caring environment.	The ethics of words is not only words directed towards the other but also the tone of the language between caregivers. This has an effect on the caring culture and makes it undignifying, where the caring environment is a part.	The ethics of words and appropriated ground values in a caring culture.
‘You must live your attitudes’.	To care according to ground values	Only when ground values are appropriated, it is possible to say these words and use them as a base for caring.	
When the dignity of older people is violated, they become silent.	Silence because of deprivation of dignity	Silence is an obvious sign that can be due to the suffering of the care recipient	Violation of dignity by ignorance or neglect in a non-caring culture
You can read the eyes of the resident…The eyes are the mirror of the soul, you can see much. Happiness and contentment can be seen. It is a competence you should have.	The art of reading the eyes.	An art of caring to confirm the other even without words.	To amplify dignity in relationships by the creative art of caring in a caring culture
